# Contemporary use of coronary artery calcium for the allocation of aspirin in light of the 2022 USPSTF guideline recommendations

**DOI:** 10.1016/j.ajpc.2022.100427

**Published:** 2022-11-03

**Authors:** Dhiran Verghese, Sanjay Manubolu, Matthew J Budoff

**Affiliations:** a Section of Advanced Cardiovascular Imaging, Division of Cardiovascular Medicine, Department of Medicine, Harbor-UCLA Medical Center, Torrance, California, USA; b Section of Cardiovascular Medicine, Department of Medicine, Lundquist Institute at Harbor UCLA Medical Center, Torrance, California, USA

**Keywords:** Aspirin, Primary prevention, Coronary artery calcium, Atherosclerotic cardiovascular disease, CCTA, Coronary artery disease, ASCVD, atherosclerotic cardiovascular disease, CAC, coronary artery calcium, CCTA, Cardiac computed tomography angiography, CVD, cardiovascular disease, GI, gastrointestinal, MACE, major adverse cardiovascular events, MI, myocardial infarction, MESA, multiethnic study of atherosclerosis, NNH, number needed to harm, NNT, number needed to treat, PCE, pooled cohort equation, TIA, transient ischemic attack, USPSTF, united States preventive services task force

## Abstract

Aspirin has been a cornerstone for primary prevention of cardiovascular disease for decades, however its use in primary prevention has been challenged in recent years. The 2022 USPSTF guidelines lowered the recommendation for the use of aspirin in primary prevention based on the recent trials that demonstrated a low to neutral benefit and an increased bleeding risk with the use of aspirin in primary prevention. However, these trials enrolled patients at a relatively low risk for atherosclerotic cardiovascular disease (ASCVD) and higher bleeding risk which could have contributed to the negative results of the trials. ASCVD prevention is ideal when therapies are personalized based on individual risk. Coronary artery calcium (CAC) score is a robust marker of atherosclerosis and reliably predicts the ASCVD risk in a graded fashion. Several studies have demonstrated the use of a CAC≥100 to identify patients who will benefit from the use of aspirin in primary prevention. Furthermore, a CAC=0 identifies patients in whom aspirin would lead to net harm. In the continuum of risk from primary to secondary prevention, CAC is likely to identify the level of risk that warrants aspirin use in patients with subclinical ASCVD. The ACC/AHA 2019 primary prevention guidelines recommend the use of CAC to reclassify risk and guide personalized allocation of statins and aspirin. Although the USPSTF has not endorsed the use of CAC in the past, given an extensive body of evidence for use of CAC to guide primary preventive therapies including aspirin, it seems reasonable to use CAC to identify the level of plaque burden at which the benefit of aspirin outweighs its risk in clinical practice and personalize theallocation of aspirin in primary prevention. Future studies and randomized trials assessing the role of preventive therapies should use CAC score for risk stratification.

## 1 Introduction

Aspirin has been the cornerstone therapy for cardiovascular disease (CVD) for decades, however its use for primary prevention of ASCVD (atherosclerotic cardiovascular disease) has been heavily debated in recent years. The 2022 USPSTF (United States preventive services task force) guidelines lowered the recommendation for aspirin in primary prevention to Grade-C for adults 40–59 years-old with *a* >10% 10-year ASCVD risk and Grade-D for adults ≥ 60 years of age. This was primarily driven by three recent trials that demonstrated neutral or only modest benefit with the use of aspirin. However, these trials do not dismiss the use of aspirin but rather highlight the need for appropriate risk stratification of individuals who are likely to derive an absolute benefit with aspirin. The USPSTF guidelines are followed by primary care clinicians across the United States making it vital to appraise the contemporary literature on this clinically relevant topic.

## 2 Current evidence

### 2.1 Historical trials in the 1900′s

Trials of aspirin in primary prevention date back to the British male doctors’ study from 1974 that showed no benefit with aspirin. This was followed by the US physician health study (PHS) that found no difference in cardiovascular death (primary endpoint) but noted a 44% reduction in myocardial infarction (MI). The primary prevention project demonstrated a 44% reduction in cardiovascular mortality and 23% reduction of cardiovascular events with no difference in all-cause mortality. The thrombosis prevention trial (TPT) showed a 32% reduction in non-fatal MI and the hypertension optimal treatment (HOT) study noted a 36% reduction in individuals hospitalized with MI. These studies, that led to the recommendation of aspirin for primary prevention, randomized patients with hypertension, diabetes, and hyperlipidemia, in an era when preventive strategies were not widely implemented.

### 2.2 Recent trials

In the aspirin to reduce risk of initial vascular events (ARRIVE) trial, 12,546 patients with estimated pooled cohort equation (PCE) risk of ∼17% were randomized to aspirin or placebo. In an intention-to-treat analysis, the composite endpoint of cardiovascular death, MI, unstable angina, stroke, and transient ischemic attack was neutral (0.84 vs. 0.88%/y, HR 0.96, 95% CI 0.81–1.13; *p* = 0.60) and there was no difference in rates of non-fatal MI. [1] The aspirin group had higher gastrointestinal bleeding but similar rates of hemorrhagic stroke. [1] In primary preventive trials, such as ARRIVE, with a long follow-up period, an intention-to-treat analysis may be biased towards a negative result which is reflected by a larger beneficial effect of aspirin noted early in the study compared with its effect later in the study. [1] Aspirin is ubiquitously available over the counter, is used for pain, inflammation, fever and is even a component of heart burn medications such as Alka seltzer. Only 61% of participants adhered to the initial randomization and such cross-contamination could have attenuated the risk reduction of aspirin. Under these circumstances, a per-protocol analysis including the absolute number of patients on aspirin is more appropriate and this analysis indicated a 47% reduction in MI (37 vs. 72 events, HR 0.53; *p* = 0.0014), mirroring results of the PHS. However, it should also be kept in mind that a per-protocol analysis could affect the balance of randomization, covariates and lead to differential exclusion of subjects with severe disease. It is noteworthy that despite the cross over, gastrointestinal bleeding, although predominantly mild, occurred more often in the aspirin group in the intention-to-treat analysis that could have resulted from an overall higher use of aspirin in the treatment compared to the placebo arm. Although ARRIVE included patients with a PCE 10-year-CVD risk of 17.3%, the actual 10-year event rates in the trial was lower, at <10%, reflecting a low-risk population, which could also have contributed to neutral cardiovascular benefit, while imparting an increased risk of gastrointestinal bleeding. (Table 1) Several protocol amendments, with expansion of study endpoints were made due to the lower-than-expected event rates initially observed. [1]

**Table 1:** Estimated and observed event rates from ARRIVE, ASPREE and ASCEND, harmonized to a 10-year ASCVD risk.

Trial	Estimated 10-year ASCVD event rate	Observed 10-year ASCVD event rate
ARRIVE	17.4%	8.6%
ASPREE	22.4%	7.8%
ASCEND	20%	12.3%

The aspirin for reducing events in the elderly (ASPREE) trial randomized patients > 70 years and found no difference in ASCVD events but noted a higher rate of major bleeding in the aspirin group. [2] The estimated event rate at randomization was 22.4 per 1000 person-years, however the observed rates were lower than half the initial estimated rate. (Table-1) [3] In elderly patients >70 years of age with a low ASCVD risk, it is likely that aspirin may not have a favorable benefit-risk ratio secondary to increased bleeding. Furthermore, the unexpected higher rates of cancer noted in the trial could have exaggerated the bleeding risk.

The effects of aspirin for primary prevention in persons with diabetes (ASCEND) trial included diabetic patients >40 years of age. Aspirin use resulted in a 12% lower rate of vascular events and a 29% higher risk of major bleeding with no difference in rates of hemorrhagic stroke and fatal bleeding. [4] The estimated risk of events at randomization was 2% per year and a 5-year follow up with 10,000 participants was planned. (Table-1) However, after the first few years of the study, reported event rates in both arms were <0.6% and the steering committee added TIA to the composite primary endpoint, increased the sample size to 15,000 and extended the follow-up to 7-years. This again highlights the extremely low ASCVD risk of the patient population included in a trail to assess the benefit of ASCVD reduction with aspirin.

In ARRIVE and ASCEND, there was an inverse trend in the efficacy outcomes, with a lower risk-reduction in patients with a higher baseline predicted risk. Cardiovascular risk is not a static feature and more aggressive interventions, with strict mitigation of risk factors are perused in patients at higher risk compared to those at baseline lower risk. Such inherent challenges could to a certain extent have been what led to the noted low relative risk reduction as mentioned above. More than 90% of patients in primary preventive trials had a risk ≤1% per year (10-year risk of <10%). In these extremely low risk patients, there is a very low or no net benefit from aspirin. At the same time, limiting aspirin for secondary prevention will miss the opportunity to prevent MI and its sequelae of downstream heart failure, arrhythmias, sudden cardiac death, and long-term mortality. There exists a continuum of risk from primary to secondary prevention and it is crucial to identify the level of risk that warrants aspirin use in patients with subclinical ASCVD. [5] There has been a paradigm shift in preventive cardiology from screening of patients with a binary (obstructive vs non-obstructive or ischemic vs non-ischemic) outcome to the identification of subclinical atherosclerosis with cardiac computed tomography (CT) and coronary CT angiography (CCTA). This has allowed for improved risk stratification of patients beyond traditional risk factors providing enhanced therapeutic guidance.

### 2.3 Coronary artery calcium – a practical approach to individualized risk assessment

Coronary artery calcium (CAC) is an excellent marker of atherosclerosis. The PCE substantially overestimates the cardiovascular risk and CAC improves the predictive value of the PCE. (6) Improved control of hypertension, diabetes, and smoking, coupled with widespread use of statins has significantly lowered the ASCVD risk since publication of the PCE, which is likely the major reason for overestimation of risk of patients in the contemporary era. CAC predicts both 5-year and 10-year ASCVD risk in a graded fashion independent of traditional risk factors. [7,8] Furthermore, CAC has proved to be superior to all other nontraditional subclinical markers of atherosclerosis for reclassification of cardiovascular risk. [9] The 2019 ACC/AHA guidelines recommend the use of CAC in borderline- and intermediate-risk patients to upgrade or downgrade risk, with a CAC ≥100 to have value in guiding the allocation of primary preventive therapies [10]. In the absence of randomized clinical trials assessing the role of CAC guided allocation of primary preventive therapies, we need to rely on data from large prospective registries and observational studies. The Multiethnic Study of Atherosclerosis (MESA) is an ongoing, prospective, sex and race balanced, observational cohort, initiated in 2000, with the goal of providing new information on detection, progression, and prognosis of subclinical cardiovascular disease. MESA emphasized the use of imaging technologies to characterize cardiovascular diseases and one of its most important legacies has been the identification of the value of CAC as a measure of subclinical atherosclerosis [11].

In a study from MESA, Miedema et al. demonstrated that participants with a CAC≥100 had a favorable risk/benefit estimation while those with a CAC=0 received net harm with the use of aspirin. [12] Furthermore, CAC ≥100 identified individuals who would potentially derive a net benefit from aspirin across all the Framingham Risk Score subgroups. [12] This study used a 2009 meta-analysis data for efficacy and safety estimates and used fixed aspirin-related bleeding risk for all the subgroups. A subsequent analysis by Cainzos-Achirica et al., applying an updated 2019 meta-analysis estimate and using observed ASCVD and observed bleeding rates in MESA, demonstrated that in individuals <70 years, free of clinically evident ASCVD, a CAC score of ≥100 identified subgroups wherein the Number needed to treat (NNT) was lower that the number needed to harm (NNH) showing an absolute benefit with the use of aspirin. [13] This was seen across the PCE risk categories in patients with a CAC ≥100 (ASCVD risk <5% NNT=300, NNH=794; ASCVD risk 5–20%-NNT=124, NNH=229; ASCVD >20%-NNT 136, NNH 256) and a CAC ≥400 (ASCVD risk <5%-NNT=100, NNH=794; ASCVD risk 5–20%-NNT=97, NNH=229; ASCVD risk >20%-NNT=107, NNH=256). [13] CAC=0 consistently identified individuals in whom aspirin therapy would lead to net harm..

In 2,384 patients with diabetes, similar simulation modeling using estimates from the CAC Consortium, Silverman et al. demonstrated that a CAC>100 reliably identified patients who benefit from the use of aspirin. However, bleeding risk was not modeled in this study. In patients with CAC>100 from the Dallas Heart Study, aspirin use was associated with a net benefit. [14,15] As expected, CAC=0 identified subjects in whom aspirin would lead to net harm. [13,14]. The margin of the ASCVD benefit to bleeding risk showed a step wise improvement with increasing CAC group categories in each of the studies.

**Fig. 1:**
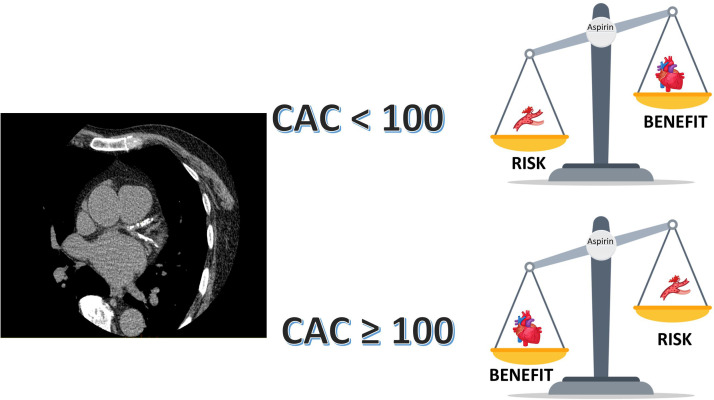
Central Figure. 1. Coronary artery calcium for allocation of aspirin in primary prevention Abbreviations: CAC: coronary artery calcium score

Although it is well known that ASCVD risk increases with CAC, there have been reports of increased bleeding risk in patients with increasing CAC scores. Higher CAC scores were associated with a higher bleeding risk in studies from MESA and the DHS. [15,16] However, the extent of bleeding risk was attenuated on multivariable adjustment [15,16]. CAC is a surrogate marker of atherosclerotic burden and there exists a correlation between CAC and other ASCVD risk factors including age, that could explain the increased bleeding noted with increasing CAC scores. However, CAC has a significantly greater magnitude for association with ASCVD events. [15,16] This is probably one of the reasons why CAC is likely to outperform risk prediction scores, as chronological age is a key driver of risk in the PCE[17] The 2019 ACC/AHA guidelines recommend the use of CAC to reclassify risk to guide personalized allocation of statins and aspirin in primary prevention. Furthermore, with the recent ACC/AHA chest pain guidelines giving a class IA recommendation for coronary CCTA in stable or acute chest pain in patients with no known coronary artery disease, it is likely that there will be increased availably of CAC scores, providing an opportunity for it to be promptly used for initiation of preventive therapies Fig. 1, [18].

## 3 Conclusion

Aspirin lowers ASCVD events in primary prevention. The cardiovascular protection of aspirin in low-risk individuals is offset by the increased bleeding risk. Achieving clinical benefit with a medication requires appropriate risk stratification, especially when there exists a potential risk such as bleeding as a side effect from the medication. The PCE overestimates ASCVD risk in the contemporary era. CAC score or modern risk calculators that include the CAC score such as the MESA and Astro-CHARM (Astronaut Cardiovascular Health and Risk Modification) risk assessment tools could be reliable gatekeepers for risk stratification and initiation of preventive therapies. In the absence of a high bleeding risk, aspirin is likely to be beneficial in individuals <70 years of age with a CAC ≥ 100 and they have an absolute net benefit with its use. CAC could potentially be used to risk stratify individuals 40–59 years of age, as well as those 60–70 years of age to personalize the use of aspirin. Although some CAC studies included participants >70 years, additional data is necessary before a recommendation for aspirin, based on CAC, can be confidently made in this population. Further studies should assess the role for CAC in risk stratifying elderly (>70 year of age) individuals to aspirin. Future randomized trials assessing the role of aspirin in primary prevention should use CAC scores for risk stratification.

## Sources of funding

None.

## Disclosures

All other authors have reported that they have no relationships relevant to the contents of this paper to disclose.

## Declaration of Competing Interest

The authors declare the following financial interests/personal relationships which may be considered as potential competing interests. None of the authors have any relationships to disclose.
